# Improved patient value after implementing same day discharge for patients undergoing pulmonary vein isolation

**DOI:** 10.1093/europace/euag155

**Published:** 2026-10-08

**Authors:** Stacey R Slingerland, Mileen R D van de Kar, Jasper Vermeer, Gijs J van Steenbergen, Daniela N Schulz, Dennis van Veghel, Lukas Dekker

**Affiliations:** Catharina Heart Centre, Catharina Hospital, P.O. Box 1350, Eindhoven 5602 ZA, the Netherlands; Catharina Heart Centre, Catharina Hospital, P.O. Box 1350, Eindhoven 5602 ZA, the Netherlands; Catharina Heart Centre, Catharina Hospital, P.O. Box 1350, Eindhoven 5602 ZA, the Netherlands; Catharina Heart Centre, Catharina Hospital, P.O. Box 1350, Eindhoven 5602 ZA, the Netherlands; Catharina Heart Centre, Catharina Hospital, P.O. Box 1350, Eindhoven 5602 ZA, the Netherlands; Catharina Heart Centre, Catharina Hospital, P.O. Box 1350, Eindhoven 5602 ZA, the Netherlands; Catharina Heart Centre, Catharina Hospital, P.O. Box 1350, Eindhoven 5602 ZA, the Netherlands; Department of Biomedical Technology, Eindhoven University of Technology, Eindhoven, the Netherlands

**Keywords:** Value-based healthcare, Patient value, Outcome measurement, Quality improvement, Pulmonary vein isolation, Same-day discharge

## Abstract

**Aims:**

Pulmonary vein isolation (PVI) is an effective intervention to treat atrial fibrillation, which is increasingly prevalent. Same-day discharge (SDD) is a strategic approach to limit procedure-related costs. This study aimed to compare healthcare utilization, costs, and clinical outcomes before and after implementation of an SDD protocol.

**Methods and results:**

This retrospective study included 2987 patients undergoing PVI between 1 January 2016 and 1 April 2021. Baseline characteristics, pre-, peri-, and post-procedure healthcare utilization and clinical outcomes were compared before (cohort A; *n* = 1379) and after (Cohort B; *n* = 1608) implementation of the SDD protocol using bivariate and multivariate analyses. show that cohort B vs. cohort A had lower all-cause 90-day mortality (0% vs. 0.3%, *P* = 0.031), no differences in re-ablation < 1 year (*P* = 0.286), as well as in complications: bleedings (*P* = 0.177), tamponade < 30 days (*P* = 0.157), phrenic nerve injury (*P* = 0.984), thromboembolic complication < 72 h (*P* = 0.212), or vascular complications (*P* = 0.290). In terms of healthcare utilization and costs, cohort B had significantly less peri-procedure hospital admission costs (€721.52 vs. €1011.78, *P* < 0.001) and less total peri- and post-procedure costs (€840.37 vs. €1152.94, *P* < 0.001). Adjusted for all baseline characteristics, the post-implementation cohort showed on average €315 lower total costs per patient.

**Conclusion:**

The implementation of the SDD protocol was associated with comparable complication rates while significantly reducing both peri- and post-procedure healthcare utilization and costs, thereby enhancing patient value. This indicates a potential margin for further cost reductions and increased efficiency.

## Graphical Abstract

**Graphical Abstract:**
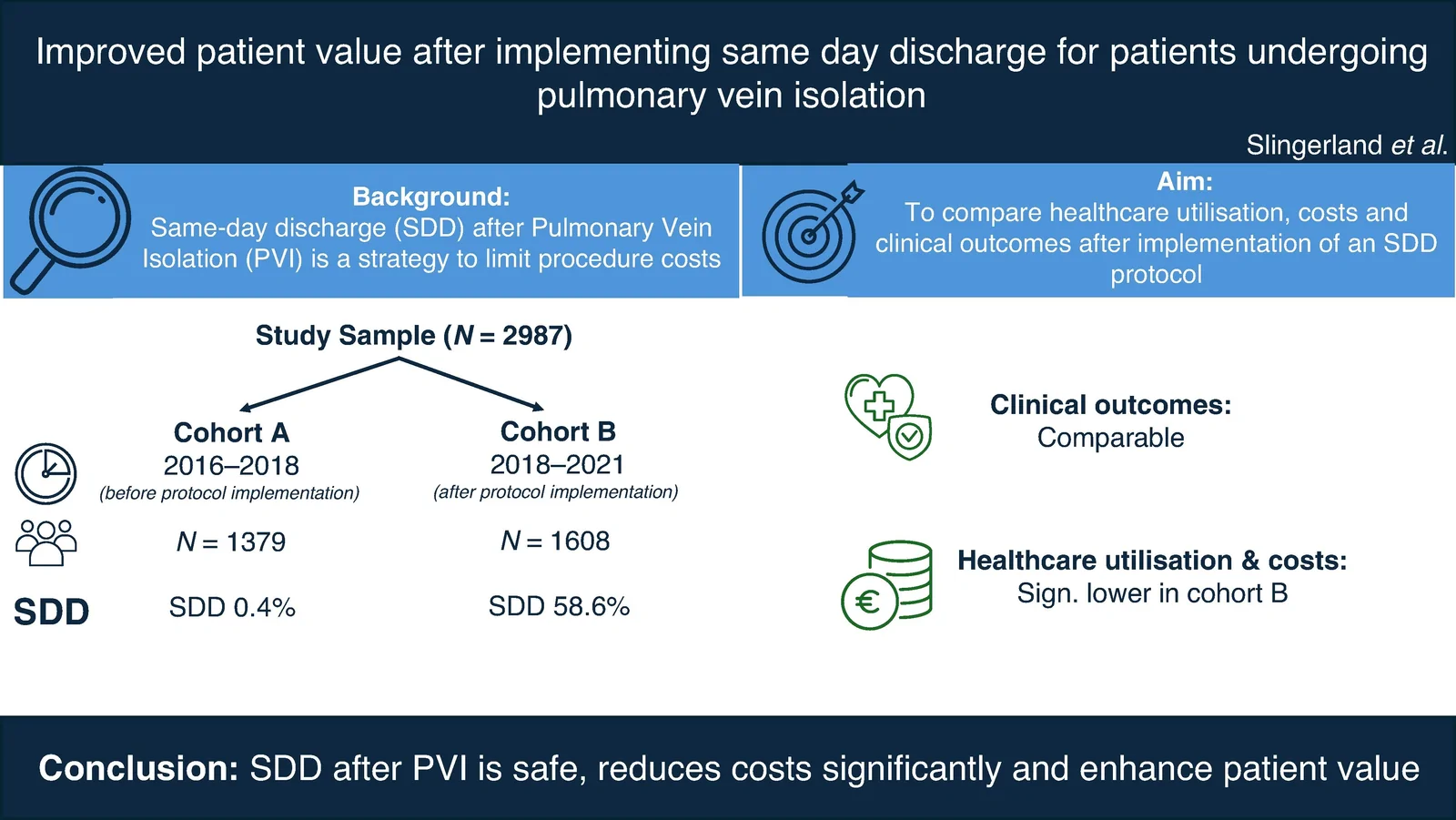

## Introduction

Atrial fibrillation (AF), the most common cardiac arrhythmia, is often treated with pulmonary vein isolation (PVI) as this is superior to antiarrhythmic drug therapy.^1–5^ PVI is an effective treatment for symptomatic paroxysmal or persistent AF, albeit resource-intensive and costly, highlighting the need for cost-effectiveness evaluations in electrophysiology and illustrating the potential of value-based healthcare approaches to optimize both patient outcomes and resource use.^2,6–8^ As the incidence and prevalence of AF, and therefore the demand for PVI, are expected to rise, it is essential to take measures to limit excessive costs.^3,4^

PVI, which started as a high-risk procedure, requiring full anaesthesia and an overnight stay (ONS), has evolved over recent years into a routinely performed intervention, increasingly allowing for same-day discharge (SDD).^1,2^ Multiple studies have demonstrated the safety and feasibility of SDD following PVI, showing no increase in short-term adverse events, complications, or readmissions compared to ONS.^8–20^ Among these, a large retrospective cohort by Deyell *et al*., including over 3000 patients reported no differences in 30-day mortality, stroke, bleeding, or hospitalization between patients discharged the same day and those who stayed overnight.^21^

However, literature on the impact of SDD after PVI on healthcare costs and healthcare consumption remains scarcely available, despite their clinical and societal relevance. In 2018, the Catharina Hospital Eindhoven (CHE) implemented a SDD protocol, replacing the ONS approach, in order to mitigate costs and improve efficiency and resource allocation.^22^ Given the substantial costs and the significant impact on healthcare activities involved in the care pathway for patients with AF, it is essential to systematically identify the patient-relevant cost drivers (PRCD) specific to this medical condition. PRCD are defined as healthcare utilization parameters that influence patient outcomes and are closely related to costs.^23^ Therefore, the primary objective of this study is to evaluate the clinical outcomes and PRCDs between two distinct cohorts: Patients treated before and after the implementation of the SDD protocol. This evaluation aims to assess if patient-relevant clinical outcomes are improved and costs reduced, in alignment with the concept of patient value, which emphasizes enhancing both clinical outcomes and costs efficiency for specific medical conditions.^24^

## Methods

### Study design

This retrospective pre–post study used real-world observational data from all patients undergoing primary PVI at CHE between 1 January 2016 and 1 April 2021. In June 2018, CHE implemented a SDD protocol as part of a broader quality improvement initiative. Patients who underwent PVI between January 2016 and May 2018 were assigned to the pre-implementation cohort (cohort A), during which ONS was standard practice. Patients treated between June 2018 and April 2021 were assigned to the post-implementation cohort (cohort B), in which the SDD protocol was applied and patients could be discharged either with SDD or after an ONS. The two cohorts therefore represent sequential patient groups undergoing the same procedure before and after the implementation of the new SDD protocol, without changes in general PVI eligibility criteria.

### Ablation and discharge protocols

During the study period, cryoballoon ablation was the most frequently employed technique for PVI, followed by radiofrequency (RF) ablation. Cryoballoon procedures were performed via unilateral femoral venous access using two sheaths (8Fr and 12Fr) for left atrial access. RF ablation involved bilateral femoral venous access, using 7Fr sheaths for coronary sinus catheter placement and two 8.5Fr transseptal sheaths for mapping and ablation, with additional arterial access for haemodynamic monitoring. Ultrasound-guided access was not standard practice. All procedures were performed under conscious sedation. The sedation protocol in our centre consisted of a combination of opioid and hypnotic drugs; esketamine, propofol, and alfentanil, which were administered according to standard practice. All procedures were performed under uninterrupted oral anticoagulation, with intravenous heparin administered to maintain an activated clotting time >300 s. Protamine was administered after retraction of catheters from the left atrium. Manual compression and a six-hour compression bandage were used for haemostasis, and groin assessment was performed by the attending physician prior to discharge.

In cohort A (pre-implementation), ONS was standard for all patients, with discharge occurring the day after the procedure. In cohort B (post-implementation), the SDD protocol was introduced, meaning this cohort includes both SDD and ONS patients. Under this protocol, patients were scheduled for SDD if the procedure ended before 3 p.m. and no (suspected) complications occurred. Patients with procedures ending after 3 p.m. were typically scheduled for ONS, as later completion limits the available observation period prior to discharge. Importantly, procedural timing was determined by standard scheduling logistics and was not based on patient characteristics or anticipated clinical risk. In addition, overnight admission could still be chosen at the operator's discretion, for example, in the case of procedural complexity or post-procedural uncertainty. As such, the decision for SDD was based primarily on procedural timing and physician judgement, rather than fixed exclusion criteria such as age, heart failure, or comorbidity. Cohort B includes patients discharged either the same day or after ONS, reflecting real-world routine practice under the improved discharge protocol.

No changes were made to the standard post-procedural follow-up protocol during the study period; any differences in monitoring reflect variations in routine outpatient practice rather than protocol revisions.

### Data collection

Data on baseline characteristics and clinical outcomes were previously collected from patient records for submission to the Dutch Heart Registration (NHR).^25^ The CHE, like all other centres in the Netherlands that provide cardiac care, participates in the NHR as part of quality improvement efforts to which all participating hospitals are committed. As such, the NHR has robust methods in place for data definitions, collection and validation whereby the acquiring of data is secured.^26,27^ Data on healthcare utilization and costs prior, during and after hospital admission were accessed through Dutch Hospital Data (DHD), which manages medical and administrative data registered by Dutch hospitals. DHD data consist of information originating from all hospitals nationwide, thus providing a complete follow-up on all included patients. Data were collected 1 year prior to 1 year after the date of intervention. Readmission data from DHD were limited to cardiovascular causes. The datasets contained patient identifiers and were handled in accordance with national privacy regulations and institutional protocols.

### Patient characteristics

For the present study, the following baseline characteristics were included: sex, age, body mass index, renal function using estimated glomerular filtration rate (eGFR) based on the MDRD-formula, left ventricular ejection fraction (LVEF), left atrial volume index (LAVI), AF-type (paroxysmal, persistent, or longstanding persistent), preoperative mitral valve regurgitation, prior ablation, CHA2DS2-VASc score, catheter ablation method (radiofrequency or Cryo-balloon), and additional catheter ablation.

### Clinical outcomes

The following clinical outcomes, which are also standard parameters within NHR, were used: all-cause mortality within 90 days, bleeding complications during hospital stay, thromboembolic complication <72 h, vascular complication within 30 days, cardiac tamponade within 30 days, phrenic nerve injury during admission and reintervention without one year.^28^ Mortality data, including all-cause mortality, were obtained through verification of the regional municipal administration registration (*Basisregistratie Personen*). Bleeding complication during hospital stay is defined according to the Bleeding Academic Research Consortium (BARC), including BARC type 2 or higher, occurring during the hospitalization related to the intervention (excluding cardiac tamponade). The definition of thromboembolic complication within 72 h is the occurrence of an ischaemic cerebrovascular accident, peripheral embolism (a thromboembolic event outside the brain, heart, eyes and lungs) or pulmonary embolism, within 72 h of the intervention. Furthermore, vascular complications within 30 days were defined according to the Valve Academic Research Consortium-2 definitions. A cardiac tamponade within 30 days is defined as the presence of >5 mm of pericardial fluid with or without clinical and/or echocardiographic signs of tamponade that occurs within 30 days of the intervention and requires intervention. Last, phrenic nerve injury is defined as failure of the phrenic nerve to function at least 24 h after the current intervention. All variable definitions are aligned with the definitions of European Society of Cardiology standards.^26,29,30^

### Patient-relevant cost drivers

The multidisciplinary methodology introduced by Van Steenbergen and colleagues (2023) was used to select and assess the patient-relevant cost drivers to analyse post-procedure healthcare utilization. In short, cost drivers were selected using a systematic stepwise approach performed by researchers, data analysts, physicians and patient representatives.^23^ The first step was to create a comprehensive list of all healthcare activities involved with a specific medical intervention. Second, the cumulative effect of each activity was calculated by adding the quantity and the price. Activities of minimal cost or negligible occurrence were excluded. Third, healthcare activities were ranked by physicians according to their ability to influence activity volume or associated costs. Finally, patient representatives ranked the care activities on patient relevance. This resulted in a selection of 7 patient-relevant cost drivers: short stay in ward < 1 day, nursing day, emergency department visits (ED), outpatient clinic visits, electrical cardioversion (ECV), Holter monitor and electrocardiogram (ECG). Short stay in ward <1 day, was defined as a form of nursing care that lasts several hours, provided in conjunction with a specialized medical examination or treatment on the same day, where the care is administered in a day-care unit. A nursing day was defined as a calendar day of nursing care provided on a ward equipped for nursing, which was part of a longer period of nursing care that includes at least one ONS. Prior healthcare utilization covered the moment a patient was placed on the waiting list for the PVI procedures, until hospital admission for the procedure. Prior healthcare utilization was included to determine if there were any differences between the cohorts before the intervention, helping to identify potential confounding factors. Therefore, prior healthcare utilization was displayed as part of the baseline characteristics. Peri-intervention utilization and costs encompassed healthcare utilization provided during hospital admission, measured from the day of the patient's admission until the day of discharge, including those admitted and discharged on the same day, but excluding the costs of the procedure itself. Post-intervention utilization is calculated from date of intervention up to one year of follow-up and is displayed for three-time intervals: less than 30 days, less than 120 days, and less than 1 year post-procedure. Post-intervention utilization was examined to compare costs between both cohorts, assessing the effects of relocation or delay of care after SDD compared to ONS. Total costs were calculated as a sum of peri-procedure costs and post-procedure costs.

### Statistical analyses

The patient characteristics, that were missing at random and missing less than 35%, were imputed using ten iterations and ten imputations using both baseline characteristics and outcomes as predictors. An overview of all variables and the rate of completeness can be found in Supplementary material online, *Table S1*. The statistical analyses involved the depiction of baseline characteristics and clinical outcomes as absolute and relative frequencies for categorical data, and as the median with interquartile range (IQR) for continuous data. The cost drivers were presented using the number of unique patients utilizing the specific cost driver (*n*(%)) and by using absolute and relative frequencies to indicate how many times each cost driver was utilized. In terms of costs, the median with IQR, mean and sum of the cost-drivers were displayed. The pre- and post-quality improvement cohorts were analysed separately, and a comparison between the two cohorts for all variables was conducted using the Mann–Whitney *U* test or Chi-square test, as appropriate. Following current guidelines on the imputation of data by The Dutch Journal of Medicine (2013) and BMC Medical Research Methodology (2017), missing values of baseline

characteristics were imputed.^31,32^ Last, a multiple regression model was employed to analyse differences in the total costs between the two cohorts, adjusting for baseline characteristics and pre-procedure healthcare utilization. A two-tailed *P*-value <0.05 was considered statistically significant. All statistical analyses were performed in IBM SPSS version 29 or using R-studios version 4.3.1 (2023-06-16). All analyses were conducted within a secure environment with access restricted to authorized researchers only.

### Radar chart

To visualize the selected categories of clinical outcomes and cost drivers, a radar chart was employed. The model highlights distinctions across cohorts, such as pre- and post-implementation of quality improvement project or healthcare protocol.^23^ Mean costs were plotted on the radar chart. Each cohort's clinical outcomes and cost drivers were plotted, and the data points for each cohort were connected with lines. Due to variations in the ranges of clinical outcomes and cost drivers (e.g. low complication rate and high costs), the maximum values per axis were selected to ensure that the lines of the pre- and post-quality improvement cohort ran within the range of the chart. Statistically significant differences were indicated on the chart. The radar chart was constructed using POWER-BI, a Microsoft software tool.

## Results

A total of 2987 patients undergoing PVI were included; 1379 patients in cohort A (46.1%) and 1608 patients in cohort B (53.9%). *Table 1* displays the baseline and procedural characteristics of both cohorts. Patients undergoing PVI in cohort B compared to cohort A have a statistically significantly higher eGFR (median respectively 73.6 and 70.1, p0.013) and lower LVEF (83.2% vs. 87.9% has LVEF>50, *P* = 0.002). In addition, significantly more patients in cohort B had prior ablations (29.7% vs. 21.6%, *P* < 0.001), longstanding persistent AF (2.2% vs. 0.5%), *P* < 0.001), point-by-point RF (28.2% vs. 20.3%, *P* < 0.001), and more additional catheter ablations (1.6% vs. 0.7%, *P* < 0.001). Furthermore, prior to the index-ablation, patients in cohort B had statistically significantly less often 1 or more nursing days (respectively 0.7% vs. 3.5% *P* < 0.001), but more often 1 or more outpatient clinic visits (18.2% vs. 11.5%, *P* < 0.001), ECV (2.8% vs. 2.0%, *P* = 0.289), and ECG (9.3% vs. 4.5%, *P* < 0.001). Last, in cohort A, 5 patients (0.4%) had a PVI in SDD compared to 942 patients (58.6%) in cohort B.

**Table 1:** Baseline characteristics for the pre and post quality improvement cohorts

Baseline characteristic	Cohort A (*n* = 1379)	Cohort B (*n* = 1608)	*P*-value
Male, *n (%)*	930 (67.4)	1081 (67.5)	0.982
Age, years, *median (IQR), n(%)*	64 (59–70)	64 (58–71)	0.042
< 45	63 (4.6)	75 (4.7)	0.112
45–60	413 (29.9)	419 (26.1)	
60–75	780 (56.5)	950 (59.1)	
>75	124 (9.0)	164 (10.2)	
BMI, kg/m^2^, *median (IQR)*	26.9 (23.7–29.6)	26.7 (24.6–29.7)	0.313
LVEF, *n (%)*			0.002
LVEF > 50	1106 (87.9)	1315 (83.2)	
LVEF 30–50	140 (11,1)	240 (15.2)	
LVEF < 30	12 (1,0)	25 (1.6)	
eGFR, mL/min, *median (IQR)*	70.1 (58.5–81.8)	73.6 (64.5–83.6)	0.013
LAVI, mL/cm^2^ *median (IQR)*^a^	38 (28.5–53.0)	36 (30–45)	0.711
Preoperative mitral valve regurgitation, *n (%)*			0.736
None/mild	1024 (92.1)	1456 (92.0)	
Moderate	84 (7.5)	117 (7.4)	
Severe	4 (0.4)	9 (0.6)	
CHA_2_DS_2_-VASc *median (IQR)*	1 (1–3)	2 (1–3)	0.980
Prior ablation, *n (%)*	283 (21.6)	477 (29.7)	<0.001
Type AF, *n (%)*			<0.001
Paroxysmal	908 (70.1)	1072 (66.7)	
Persistent	381 (29.4)	501 (31.2)	
Longstanding persistent	6 (0.5)	35 (2.2)	
Additional catheter ablation, *n (%)*	9 (0.7)	26 (1.6)	0.015
Catheter ablation method, *n (%)*			<0.001
RF (point-by-point or PVAC)	269 (20.3)	455 (28.2)	
Cryo-balloon	1043 (79.4)	1152 (71.6)	
Pre-intervention healthcare utilization			
Short stay in ward < 1 day *n (%)*			0.966
0	1323 (95.0)	1545 (96.1)	
1	52 (3.8)	59 (3.7)	
≥ 2	4 (0.3)	4 (0.2)	
Nursing day, *n*(%)			<0.001
0	1317 (95.4)	1587 (98.7)	
1	63 (4.6)	20 (1.2)	
≥ 2	0 (0.0)	1 (0.1)	
ED visit *n*(%)			0.684
0	1272 (92.2)	1495 (93.0)	
1	72 (5.2)	73 (4.5)	
≥ 2	35 (2.5)	40 (2.5)	
Outpatient clinic visit *n*(%)			<0.001
0	1203 (87.29)	1236 (76.9)	
1	159 (11.5)	293 (18.2)	
≥ 2	17 (1.2)	79 (4.9)	
ECV *n*(%)			0.289
0	1349 (97.8)	1558 (96.9)	
1	27 (2.0)	45 (2.8)	
≥ 2	3 (0.2)	5 (0.3)	
Holter monitor *n*(%)			0.057
0	1375 (99.7)	1592 (99.0)	
1	4 (0.3)	15 (0.9)	
≥ 2	0 (0)	1 (0.1)	
ECG, *n*(%)			<0.001
0 ECG	1273 (92.3)	1358 (84.5)	
1 ECG	62 (4.5)	149 (9.3)	
≥ 2 ECG	44 (3.2)	101 (6.3)	

^a^LAVI (left atrial volume index) has missing values: 1337 missing values (96.9%) in the cohort A and 927 (57.6%) in cohort B. BMI; body mass index, LVEF; left ventricular ejection fraction. AF; atrial fibrillation, ED; emergency department, ECV; electrical cardioversion, ECG; electrocardiogram.

*Table 2* shows the differences in clinical outcomes between the two cohorts. Cohort B, compared to the cohort A, has a statistically significantly lower 90-day mortality rate (0% respectively 0.3%, *P* = 0.031). There are no statistically significant differences between both cohorts in terms of complications and re-ablations.

**Table 2:** Clinical outcome for the pre and post cohort

Clinical outcome	Cohort A (*n* = 1379)	Cohort B (*n* = 1608)	*P*-value
Readmissions <30 days, *n (%)*	29 (2.1)	30 (1.9)	0.642
Mortality < 90 days, *n (%)*	4 (0.3)	0 (0.0)	0.031
Re-ablation < 1 year, *n (%)*	254 (18.4)	272 (16.9)	0.286
Bleeding complications during admission, *n (%)*	6 (0.5)	14 (0.9)	0.177
Tamponade < 30 days, *n (%)*	10 (0.8)	6 (0.4)	0.157
Phrenic nerve injury, *n (%)*	14 (1.1)	17 (1.1)	0.984
Thromboembolic complication < 72 h, *n (%)*	7 (0.5)	4 (0.2)	0.212
Vascular complications < 30 days, *n (%)*	0 (0.0)	3 (0.2)	0.290

*Table 3* displays the post-intervention healthcare utilization and costs for cohort A and B. In terms of the one year post-intervention healthcare utilization, cohort B, compared to cohort A, had statistically significantly fewer patients with nursing days (11.9% vs. 20.6%, *P* < 0.001), ED visits (22.5% vs. 26.5%, *P* = 0.012), ECV (8.5% vs. 10.9%, *P* = 0.021), Holter monitors (19.3% vs. 22.5%, *P* = 0.031), and ECGs (73.4% vs. 91.4%, *P* < 0.001). However, there were significantly more patients with short admissions <1 day (17.8% vs. 12.5%, *P* < 0.001). Supplementary material online, *Table S2* materials provides a comprehensive overview of the frequency and distribution of patient-relevant cost drivers for both cohorts.

**Table 3:** Post-intervention healthcare utilization between cohort A and B

Patient-relevant cost-driver post-intervention	< 30 days			< 120 days			< 1 year		
	Cohort A (*n* = 1379)	Cohort B (*n* = 1608)	*P*-value	Cohort A (*n* = 1379)	Cohort B (*n* = 1608)	*P*-value	Cohort A (*n* = 1379)	Cohort B (*n* = 1608)	*P*-value
Short stay in ward, yes, *n* (%)	14 (1.0%)	27 (1.7%)	0.120	65 (4.7%)	115 (7.2%)	0.005	173 (12.5%)	287 (17.8%)	<0.001
Nursing day, yes, *n*(%)	29 (2.1%)	34 (2.1%)	0.983	72 (5.2%)	67 (4.2%)	0.173	284 (20.6%)	192 (11.9%)	<0.001
ED^a^ visit, yes, *n* (%)	160 (11.6%)	150 (9.3%)	0.042	249 (18.1)	243 (15.1)	0.031	365 (26.5)	362 (22.5)	0.012
Outpatient clinic visit, yes, *n* (%)	160 (11.6%)	182 (11.3%)	0.808	1277 (92.6%)	1451 (90.2%)	0.022	1351 (98.0%)	1569 (97.6%)	0.467
ECV^a^, yes, *n* (%)	28 (2.0%)	24 (1.5%)	0.262	86 (6.2%)	80 (5.0%)	0.134	151 (10.9%)	136 (8.5%)	0.021
Holter monitor, yes, *n* (%)	8 (0.6%)	3 (0.2%)	0.077	93 (6.7%)	114 (7.1%)	0.711	310 (22.5%)	310 (19.3%)	0.031
ECG^a^, yes, *n*(%)	122 (8.8%)	205 (12.7%)	<0.001	1132 (82.1%)	912 (56.7%)	<0.001	1261 (91.4%)	1181 (73.4%)	<0.001

^a^ED, emergency department; ECV, electrical cardioversion; ECG, electrocardiogram.

*Table 4* presents the costs per cost driver between cohort A and B. In cohort B, the costs for ED visits <30 days are statistically significantly lower compared to cohort A (on average €37.07 vs. €50.11, respectively, *P* = 0.036) while the costs for ECGs < 30 days are significantly higher (€7.56 vs. €4.93, respectively, *P* < 0.001). Per-procedure hospital admission costs were statistically significantly lower in cohort B (on average €721.52) compared to cohort A (on average €1011.78, *P* < 0.001), just as the total per- and post-procedure costs (on average €840.37 and €1152.94 *P* < 0.001, respectively).

**Table 4:** Post-intervention costs per cost driver between cohort A and B

PRCD	Cohort A (n = 1379)			Cohort B (n = 1608)			P-value
	No. of patients (%)	Median €, (IQR)	Mean €	No. of patients (%)	Median € (IQR)	Mean €	
Short stay in ward < 30 days	14 (1.0%)	0 (0–0)	4.82	27 (1.7%)	0 (0–0)	7.97	0.120
Nursing day < 30 days	29 (2.1%)	0 (0–0)	52.36	34 (2.1%)	0 (0–0)	40.96	0.985
ED^a^ visit <30 days	160 (11.6%)	0 (0–0)	50.11	150 (9.3%)	0 (0–0)	37.06	0.036
Outpatient clinic visit <30 days	160 (11.6%)	0 (0–0)	13.03	182 (11.3%)	0 (0–0)	12.20	0.745
ECV^a^ < 120 dagen	86 (6.2%)	0 (0–0)	7.17	80 (5.0%)	0 (0–0)	5.71	0.132
Holter monitor < 120 days	93 (6.7%)	0 (0–0)	8.73	114 (7.1%)	0 (0–0)	7.49	0.777
ECG^a^ < 30 days	122 (8.8%)	0 (0–0)	4.93	205 (12.7%)	0 (0–0)	7.56	<0.001
Peri-procedure hospital admission costs	Sum in euros	975.7 (975.7–975.7)	1011.78	Sum in euros	474.7 (474.7–975.7)	721.52	<0.001
Total costs peri- and post-procedure	Sum in euros	795.7 (975.7–1077.7)	1152.94	Sum in euros	576.7 (474.7–975.7)	840.37	<0.001

^a^ED, emergency department; ECV, electrical cardioversion; ECG, electrocardiogram.

Last, Supplementary material online, *Table S3* shows the multiple Regression for the total per- and post-procedure costs between Cohort A and B while adjusting for all baseline characteristics. The cohort variable is statistically significant after adjusting for patient characteristics and pre-procedure healthcare utilization (*B* = −316.06, *P* < 0.001). Transitioning from cohort A to cohort B is associated with a reduction of approximately €316 in total costs (peri and post-procedure), ceteris paribus.

*Figure 1* shows the radar chart displaying a selection of clinical outcomes and patient-relevant cost drivers for the pre- and post-cohort. The key findings from *Tables 1 to 4* are summarized, offering a short overview of the differences between cohorts A and B at a glance.

**Figure 1:**
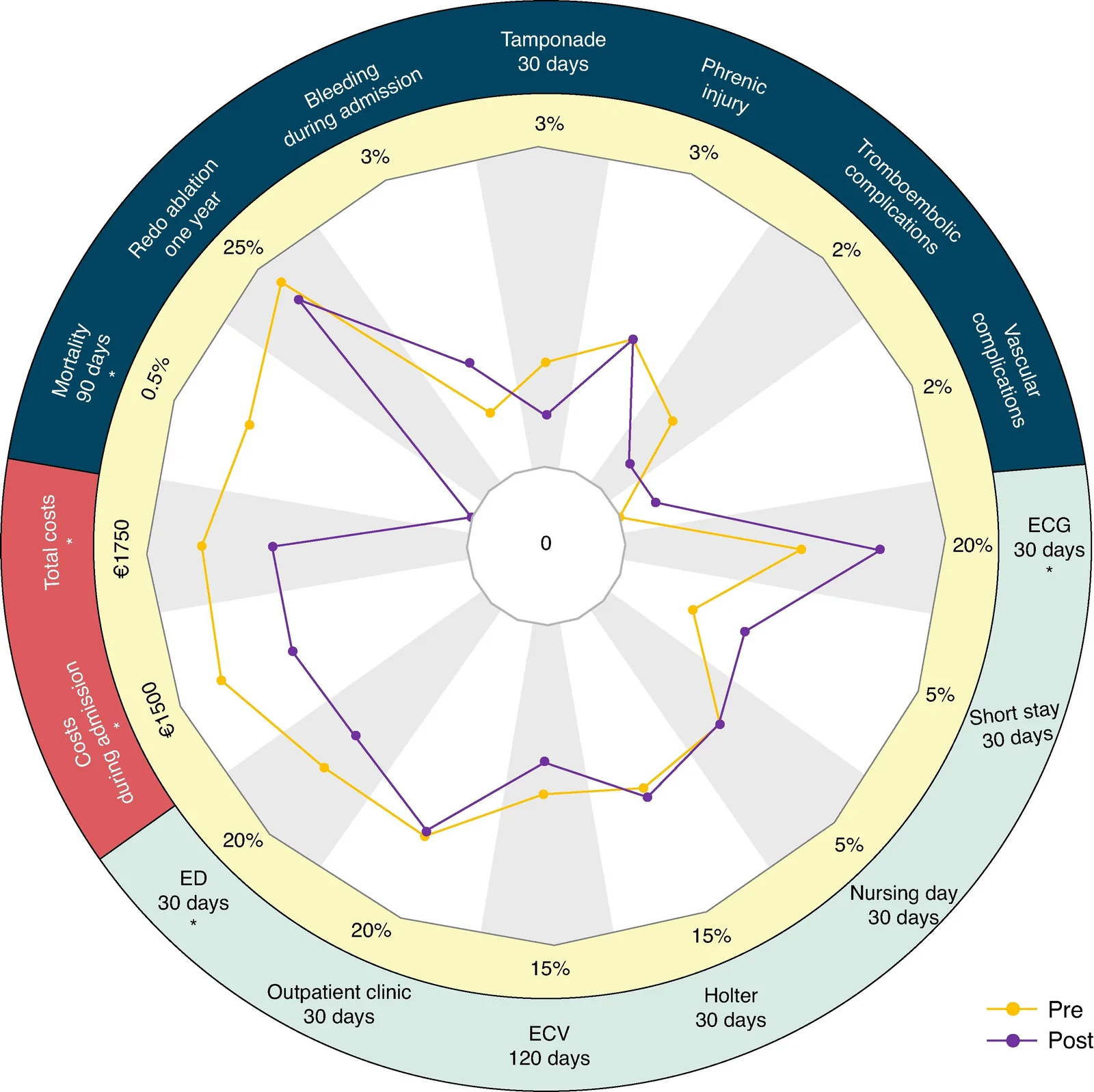
Clinical outcomes and patient-relevant cost drivers for cohort A and cohort B.

Parameters with an * present a statistically significant difference.

## Discussion

The objective of this study was to evaluate the clinical outcomes and patient-related cost drivers in two distinct cohorts of patients undergoing PVI AF, before and after implementation of a SDD protocol. The introduction of the SDD protocol was associated with lower 90-day mortality, comparable complication rates, and lower healthcare utilization and costs.

Fewer nursing days and a lower 90-day mortality rate were observed in cohort B following implementation of the SDD protocol. The decline in nursing days is an anticipated effect following the SDD protocol, as patients are discharged the same day or after a shorter observation period. In contrast, the difference in 90-day mortality cannot be fully explained and should be interpreted with caution given the low absolute number of events and the potential for selection bias. While baseline characteristics were largely similar between cohorts, cohort B included a slightly higher proportion of patients with reduced LVEF, longstanding persistent AF, prior ablations, and additional catheter ablation procedures. These cohort differences may reflect evolving referral patterns and increasing clinical confidence in offering ablation to more complex patients, including those with advanced comorbidities or older age. The observed difference in mortality and healthcare use should be interpreted within the broader context of care pathway optimization, rather than as effects attributable solely to the discharge strategy. The other clinical findings on the safety of this study are consistent with several other studies, supporting the broader applicability of SDD protocols.^8–20^

The study further demonstrated that SDD implementation was not associated with higher resource consumption or redistribution of healthcare utilization. In cohort B, significantly lower healthcare use was observed during both the peri- and post-procedural phases, resulting in lower total costs. In terms of hospital admissions, cohort B had a higher frequency of short-stay ward episodes but a markedly lower number of nursing days. This pattern suggests a shift towards shorter, more focused hospital encounters rather than prolonged or repeated admissions. While the overall number of hospital presentations remained stable, their nature changed from extended stays and multiple visits in cohort A to shorter, more efficient episodes in cohort B. This shift may reflect evolving hospital workflows during the study period, including growing capacity pressures and an increased emphasis on streamlined care delivery. These findings highlight the importance of considering both clinical outcomes and care utilization when implementing new care pathways.

Cohort B was associated with lower total healthcare costs, primarily driven by decreased resource use during admission, facilitated by earlier discharge under the SDD protocol. The observed differences occurred without any indication of compromised safety or clinical outcomes, indicating improved patient value and potential for sustainable resource use. It is important to note that not all patients in the post-implementation cohort were discharged the same day. ONS remained appropriate in a substantial proportion of cases due to procedural timing or clinical judgement. This partial uptake reflects real-world implementation and may have moderated the overall magnitude of cost savings. By reducing unnecessary ONSs, SDD protocols can support more efficient and patient-centred care. Analysis of post-procedural utilization patterns may further inform the optimization of discharge strategies.

These findings are consistent with recent European evidence. A meta-analysis by Zylla *et al*.^33^ confirmed that SDD after PVI is associated with low rates of complications, rehospitalization, and mortality. Similarly, Espinosa *et al*.^34^ demonstrated that nurse-led SDD protocols can be implemented safely and effectively in routine care. Despite this, a recent survey indicated that SDD is still applied in only a minority of European centres, highlighting a gap between emerging evidence and current practice.^35^

Further improvements in the SDD pathway may be achieved through individualized discharge planning and refined risk stratification. A personalized approach to post-ablation follow-up could support more efficient resource allocation while maintaining patient safety and satisfaction.

### Strengths and limitations

This retrospective observational study employs a practice-based approach to cover over 80% of the actual costs associated with the procedure.^23^ By doing so, it offers practical insights into the actual expenses incurred in the real world. The radar chart provides a comprehensive visual representing the various aspects of patient value in a single view. The utilization of DHD data ensures comprehensive follow-up information for all patients, thereby minimizing the risk of underestimating healthcare utilization, such as post-procedural ED visits. In addition, it employs real-world data of clinical outcomes of all patients who underwent PVI in the study period, which ensures a recent and proportionate representation.

One of the limitations of this study is the potential for bias to be introduced by the retrospective nature of the research. The improved experience and technology used in the most recent cohort (B) could have led to better outcomes that are not directly attributable to the SDD protocol. However, the same primary ablation technique was used in both cohorts, reducing chances of bias. In addition, the time to the procedure was not considered, which precluded adjustment for any additional healthcare utilization that may have occurred during an extended waiting period.

Moreover, due to the limited dataset, relevant biomedical parameters could not be included in the analysis, and detailed information on individual observation time in hours was not available. Additionally, some baseline characteristics had missing values, requiring imputation. However, for variables like LAVI, which had over 96% missing data, imputation was not feasible, leading to exclusion from this study.

In addition, previous studies have suggested that the use of general anaesthesia may be associated with fewer repeat ablations, although these findings were derived from cohorts undergoing RF ablation.^36,37^ In contrast, the majority of patients in our cohort underwent cryoballoon ablation, a technique that is less vulnerable to minor patient movements, which makes conscious sedation a safe and feasible alternative in this setting.^37–41^

Furthermore, a notable limitation of this study is the lack of detailed information regarding the reasons why patients in cohort B were not discharged on the same day. While the SDD protocol was applied consistently, individual-level data on the clinical or procedural considerations underlying the decision for ONS were not recorded. It therefore remains unclear whether these cases were due to a higher suspicion of complication, because more patients in Cohort B underwent PVI after 3 p.m., or other factors. Understanding the specific reasons or selection criteria for ONSs could potentially help inform and enhance the SDD protocol.

Another restraint of this study is that the extrapolation of our results might be limited. The study was conducted in a high-volume, tertiary heart centre, and its applicability to low-volume or less experienced centres, or to centres predominantly performing other procedural techniques, is uncertain. Furthermore, differences in socioeconomic status, healthcare access, and patient health status mean that the atrial fibrillation population in the Netherlands may not be fully representative of populations in other countries, potentially limiting the generalizability of our findings.

In accordance with the SDD protocol, further refinement of risk stratification on a patient-level could be the next logical step in order to improve healthcare utilization and expenditure in the PVI-care pathway even more. This personalized approach could help identifying patients who would benefit most from tailored post-ablation follow-up strategies, thus maximizing resource allocation and enhancing overall efficiency in patient care delivery.

## Conclusion

This retrospective study demonstrates that implementing an SDD protocol following PVI is associated with lower mortality, similar clinical outcomes, and reduced healthcare costs compared to an ONS protocol. Notably, the lower healthcare utilization observed during the initial hospital admission did not result in a subsequent reallocation or delay in healthcare utilization post-procedure. Considering the observational design, these findings suggest that the SDD protocol is not only a safe and effective alternative but also a cost-efficient approach that may enhance patient value by optimizing clinical outcomes and lower resource utilization.

## Supplementary Material

euag155_Supplementary_Data

## Data Availability

The data underlying this study are not publicly available but may be obtained upon reasonable request through the Netherlands Heart Registration (NHR) and Dutch Hospital Data (DHD), subject to their data access policies and approval procedures.
