# Supplementary material for: Improved patient value after implementing same day discharge for patients undergoing pulmonary vein isolation
Source: Europace. 2026 Oct 8;28(10):euag155. doi: 10.1093/europace/euag155 (PMC13646612; doi:10.1093/europace/euag155)
Supplement: Supplementary file 1 [file euag155_supplementary_data.docx]

**Supplementary Materials**

***Table 1 Completeness of baseline variables for cohort A and cohort B***

| **Baseline variable** | **Total (Valid N, %)** | **Cohort A (Valid N, %)** | **Cohort B (Valid N, %)** |
| --- | --- | --- | --- |
| **Sex** | 2,981 (99.8%) | 1,379 (100%) | 1,602 (99.6%) |
| **Age** | 2,987 (100%) | 1,379 (100%) | 1,608 (100%) |
| **BMI** | 2,870 (96.1%) | 1,268 (91.9%) | 1,602 (99.6%) |
| **LVEF** | 2,771 (92.8%) | 1,191 (86.4%) | 1,580 (98.3%) |
| **LAVI** | 724 (24.2%) | 43 (3.1%) | 681 (42.4%) |
| **Preoperative mitral valve regurgitation** | 2,694 (90.2%) | 1,112 (80.6%) | 1,582 (98.4%) |
| **CHA2DS2-VASc** | 2,885 (96.6%) | 1,282 (92.9%) | 1,603 (99.7%) |
| **Prior ablation** | 2,921 (97.8%) | 1,313 (95.2%) | 1,608 (100%) |
| **Additional catheter ablation** | 2,987 (100%) | 1,379 (100%) | 1,608 (100%) |
| **Catheter ablation method** | 2,921 (97.8%) | 1,313 (95.2%) | 1,608 (100%) |
| **Type AF** | 2,902 (97.2%) | 1,294 (93.8%) | 1,608 (100%) |

*BMI; body mass index, LVEF; left ventricular ejection fraction. LAVI; left atrial volume index, AF; atrial fibrillation.*

***Table 2 Post-intervention healthcare utilisation between cohort A and B***

| **Patient-relevant cost-driver post-intervention** | **< 30 days** | | | **< 120 days** | | | **< 1 year** | | |
| --- | --- | --- | --- | --- | --- | --- | --- | --- | --- |
|  | **Pre-cohort (N= 1,379)** | **Post-cohort (N = 1,608)** | **p-value** | **Pre-cohort (N= 1,379)** | **Post-cohort (N = 1,608)** | **p-value** | **Pre-cohort (N= 1,379)** | **Post-cohort (N = 1,608)** | **p-value** |
| **Short stay in ward <1 day, N (%)** |  |  |  |  |  |  |  |  |  |
| yes, N(%) | 14 (1.0%) | 27 (1.7%) | .120 | 65 (4.7%) | 115 (7.2%) | .005 | 173 (12.5%) | 287 (17.8%) | <.001 |
| 0 | 1,365 (99.0%) | 1,581 (98.3%) | .120 | 1,314 (95.3%) | 1,493 (92.8%) | .012 | 1,206 (87.5%) | 1,321 (82.2%) | <.001 |
| 1 | 14 (1.0%) | 27 (1.7%) |  | 55 (4.0%) | 90 (5.6%) |  | 121 (8.8%) | 198 (12.3%) |  |
| ≥ 2 | n/a | n/a |  | 10 (0.7%) | 25 (1.6%) |  | 52 (3.8%) | 89 (5.5%) |  |
| **Nursing day >1 day, N(%)** |  |  |  |  |  |  |  |  |  |
| yes, N(%) | 29 (2.1%) | 34 (2.1%) | .983 | 72 (5.2%) | 67 (4.2%) | .173 | 284 (20.6%) | 192 (11.9%) | <.001 |
| 0 | 1,350 (97.9%) | 1,574 (97.9%) | .991 | 1,307 (94.8%) | 1,541 (95.8%) | .203 | 1,095 (79.4%) | 1,416 (88.1%) | <.001 |
| 1 | 2 (0.1%) | 3 (0.2%) |  | 4 (0.3%) | 9 (0.6%) |  | 6 (0.4%) | 7 (0.4%) |  |
| 2 | 9 (0.7%) | 11 (0.7%) |  | 39 (2.8%) | 31 (1.9%) |  | 176 (12.8%) | 101 (6.3%) |  |
| ≥3 | 18 (1.3%) | 20 (1.2%) |  | 29 (2.1%) | 27 (1.7%) |  | 102 (7.4%) | 84 (5.2%) |  |
| **ED visit, N (%)** |  |  |  |  |  |  |  |  |  |
| yes, N (%) | 160 (11.6%) | 150 (9.3%) | .042 | 249 (18.1) | 243 (15.1) | .031 | 365 (26.5) | 362 (22.5) | .012 |
| 0 | 1,220 (88.4) | 1,458 (90.7) | .046 | 1,131 (82.0) | 1,365 (84.9) | .025 | 1,014 (73.5%) | 1,246 (77.5%) | .002 |
| 1 | 117 (8.5) | 120 (7.5) |  | 160 (11.6) | 173 (10.8) |  | 176 (12.8%) | 208 (12.9%) |  |
| ≥2 | 43 (3.1) | 30 (1.9) |  | 89 (6.4) | 70 (4.4) |  | 189 (13.7%) | 154 (9.6%) |  |
| **Outpatient clinic visit, N (%)** |  |  |  |  |  |  |  |  |  |
| yes, N (%) | 160 (11.6%) | 182 (11.3%) | .808 | 1277 (92.6%) | 1451 (90.2%) | .022 | 1351 (98.0%) | 1569 (97.6%) | .467 |
| 0 | 1,219 (88.4%) | 1,426 (88.7%) | .734 | 102 (7.4%) | 157 (9.8%) | .134 | 28 (2.0%) | 39 (2.4%) | .286 |
| 1 | 146 (10.6%) | 161 (10.0%) |  | 897 (65.0%) | 991 (61.6%) |  | 234 (17.0%) | 286 (17.8%) |  |
| 2 | 10 (0.7%) | 18 (1.1%) |  | 254 (18.4%) | 318 (19.8%) |  | 441 (32.0%) | 465 (28.9%) |  |
| 3 | 2 (0.1%) | 2 (0.1%) |  | 82 (5.9%) | 90 (5.6%) |  | 310 (22.5%) | 350 (21.8%) |  |
| ≥4 | 2 (0.1%) | 1 (0.1%) |  | 44 (3.2%) | 52 (3.2%) |  | 366 (26.5%) | 468 (29.1%) |  |
| **ECV, N (%)** |  |  |  |  |  |  |  |  |  |
| yes, N (%) | 28 (2.0%) | 24 (1.5%) | .262 | 86 (6.2%) | 80 (5.0%) | .134 | 151 (10.9%) | 136 (8.5%) | .021 |
| 0 | 1,351 (98.0%) | 1,584 (98.5%) | .408 | 1,293 (93.8%) | 1,528 (95.0%) | .310 | 1,228 (89.1%) | 1,472 (91.5%) | .022 |
| 1 | 27 (2.0%) | 22 (1.4%) |  | 75 (5.4%) | 71 (4.4%) |  | 107 (7.8%) | 107 (6.7%) |  |
| ≥2 | 1 (0.1%) | 2 (0.1%) |  | 11 (0.8%) | 9 (0.6%) |  | 44 (3.2%) | 29 (1.8%) |  |
| **Holter monitor, N (%)** |  |  |  |  |  |  |  |  |  |
| yes, N (%) | 8 (0.6%) | 3 (0.2%) | .077 | 93 (6.7%) | 114 (7.1%) | .711 | 310 (22.5%) | 310 (19.3%) | .031 |
| 0 | 1,371 (99.4%) | 1,605 (99.8%) | .177 | 1,286 (93.3%) | 1,494 (92.9%) | <.001 | 1,069 (77.5%) | 1,298 (80.7%) | <.034 |
| 1 | 7 (0.5%) | 3 (0.2%) |  | 74 (5.4%) | 110 (6.8%) |  | 236 (17.1%) | 251 (15.6%) |  |
| ≥2 | 1 (0.1%) | 0 (0.0%) |  | 19 (1.4%) | 4 (0.2%) |  | 74 (5.4%) | 59 (3.7%) |  |
| **ECG, N(%)** |  |  |  |  |  |  |  |  |  |
| yes, N (%) | 122 (8.8%) | 205 (12.7%) | <.001 | 1132 (82.1%) | 912 (56.7%) | <.001 | 1261 (91.4%) | 1181 (73.4%) | <.001 |
| 0 ECGs | 1,257 (91.2%) | 1,403 (87.3%) | .004 | 247 (17.9%) | 696 (43.3%) | <.001 | 118 (8.6%) | 427 (26.6%) | <.001 |
| 1 ECG | 89 (6.5%) | 136 (8.5%) |  | 902 (65.4%) | 569 (35.4%) |  | 508 (36.8%) | 478 (29.7%) |  |
| 2 ECGs | 15 (1.1%) | 36 (2.2%) |  | 144 (10.4%) | 178 (11.1%) |  | 352 (25.5%) | 277 (17.2%) |  |
| ≥ 3 ECGs | 18 (1.3%) | 33 (2.1%) |  | 86 (6.2%) | 165 (10.3%) |  | 401 (29.1%) | 426 (26.5%) |  |

**ED: emergency department,* *ECV; electrical cardioversion, ECG; electrocardiogram*

*Supplementary Table 3 Multiple Regression for total per- and post-procedure costs between Cohort A and B while adjusting for baseline characteristics*

|  | **Unstandardized Coefficients** | | **T** | **Sig.** |
| --- | --- | --- | --- | --- |
| **Variables** | **B** | **Std. Error** |  |  |
| (Constant) | 947,370 | 164,803 | 5,748 | 0,000 |
| Cohort | -316,064 | 27,137 | -11,647 | 0,000 |
| Sex | 56,958 | 34,316 | 1,660 | 0,097 |
| Age | 2,070 | 1,763 | 1,174 | 0,240 |
| BMI | 3,863 | 3,438 | 1,124 | 0,261 |
| eGFR | -1,066 | 0,720 | -1,480 | 0,141 |
| Preoperative mitral valve regurgitation | 122,432 | 49,112 | 2,493 | 0,013 |
| Type AF (ref. cat. is no longstanding persistent) | -35,789 | 112,744 | -0,317 | 0,751 |
| CHA2DS2-VASc | 14,573 | 12,616 | 1,155 | 0,248 |
| Additional catheter ablation | 124,311 | 123,519 | 1,006 | 0,314 |
| Catheter ablation method | -35,215 | 31,533 | -1,117 | 0,264 |
| Pre-procedure ED visits | 130,651 | 36,341 | 3,595 | 0,000 |
| Pre-procedure short stay in ward < 1 day | 205,480 | 62,271 | 3,300 | 0,001 |
| Pre-procedure nursing day >1 day | 323,159 | 18,238 | 17,719 | 0,000 |
| Pre-procedure ECG | -52,593 | 16,669 | -3,155 | 0,002 |
| Pre-procedure Holter | 171,983 | 149,649 | 1,149 | 0,250 |
| Pre-procedure ECV | -88,794 | 77,320 | -1,148 | 0,251 |
| Pre-procedure outpatient clinic visit | -24,693 | 25,097 | -0,984 | 0,325 |
| **Model summary** |  |  |  |  |
| R^2^ | .159 |  |  |  |
| Adjusted R^2^ | .154 |  |  |  |
| F-statistic (p-value) | 32.08 (<.001) |  |  |  |
| N | 2,902 |  |  |  |

*BMI; body mass index, AF; atrial fibrillation, ED; emergency department, ECV; electrical cardioversion, ECG; electrocardiogram; eGFR; estimated glomerular filtration rate*
